# Comparison of silicone double-lumen tube and polyvinyl chloride single-lumen tube in fiberoptic tracheal intubation on a difficult airway model: a randomized controlled non-inferiority trial

**DOI:** 10.1038/s41598-023-35635-1

**Published:** 2023-05-24

**Authors:** Seyoon Kang, Yun Jeong Chae, Dae Hee Kim, Se Young Bae, Ji Young Yoo

**Affiliations:** 1 Department of Anesthesiology and Pain Medicine, Ajou University School of Medicine, 164, Worldcup-ro, Yeongtong-gu, Suwon, 16499 Republic of Korea; 2 Present Address: Abijou Hospital, Incheon, Republic of Korea

**Keywords:** Medical research, Risk factors

## Abstract

The management of patients with history or suspicion of difficult intubation can be challenging, especially in surgical procedures requiring one-lung ventilation. The ease of insertion of silicone double lumen tube (DLT) have previously been shown to be comparable to polyvinyl single lumen tube (SLT) in fiberoptic bronchoscope (FOB) tracheal intubation. Hence, in difficult airway situation, we hypothesized that the performance of insertion of silicone DLT would also be non-inferior to polyvinyl SLT in FOB intubation. We used a neck collar to mimic patients with difficult airway. 80 patients who required one-lung ventilation were enrolled in a prospective, randomized, non-inferiority trial. Patients were randomly allocated to the DLT or SLT groups (SLT with bronchial blocker). Neck collar was supplied to all patients before FOB intubation. The time of insertion for FOB, railroading, tracheal intubation, and total procedure were measured. The difficulty of railroading was evaluated in 4 grades. In the DLT group, the railroading was significantly shorter and easier comparing to the SLT group. The total procedure was also simpler and faster in the DLT group. While simulated difficult airways may not fully replicate actual difficult airways, we suggest that fiberoptic intubation with silicone DLT could be a feasible first-line option for patients with expected difficult airways requiring lung separation, unless the size of the DLT relative to the patient’s airway is problematic.

**Trial registration:** NCT03392766.

## Introduction

Dealing with patients who have a difficult airway and require lung separation remains a challenging task for anaesthesiologists. Moreover, using a double lumen tube (DLT) for lung separation is more difficult compared to the use of a single lumen tube (SLT) due to the length, width, and other noncompliant characteristics of DLT^1–3^. In this situation, it would be easier and safer to initially place an SLT using video laryngoscopy or a flexible fiberoptic bronchoscopy and then replace it with a DLT using an airway catheter exchanger technique^1,2,4^. However, the characteristics of DLT mentioned earlier could also cause railroading (advancement over the fiberoptic bronchoscope or tube exchanger) to be traumatic and difficult^5–7^.

With the development of a silicone DLT (HumanBroncho, ^®^Insung Medical, Seoul, Korea), which is softer and more flexible than the existing DLT (Rusch, Mallinckrodt, Fuji), the approach to DLT insertion in patients with difficult airways can be changed. In our previous study, the flexibility of a new silicone-based DLT has been shown to be more effective in railroading than polyvinyl chloride-based SLT^8^; thus, it could allow direct intubation with DLT over the fiberoptic bronchoscope without the need for a two-step process involving the exchange of SLT for DLT. However, this study was conducted in normal airways, and no studies for difficult airways have been reported yet.

Therefore, we evaluated the intubation performance of silicone DLT and polyvinyl chloride SLT with fiberoptic bronchoscope in patients who reproduced a difficult airway with a semi-rigid neck collar.

## Methods

This was an open-label, single-centre, randomized controlled non-inferiority trial with two parallel groups. This study was approved by the Ajou Institutional Review Board (AJIRB-DEV-OBS-17-247; date of registration: 30/11/2017) and was registered at ClinicalTrials.gov (NCT03392766; date of registration: 08/01/2018). All the study procedures were performed in accordance with the relevant guidelines and regulations. Written informed consent for the study was obtained from each patient participating in the study. Patients aged 19–75 years with an American Society of Anaesthesiologists physical status of 1 and 2 who were scheduled to undergo elective thoracic surgery requiring one-lung ventilation (OLV) were enrolled in this study. Patients were excluded if they met one of the following criteria: (1) had an anatomical anomaly or intraluminal mass in the upper airway tract; (2) had a history of gastroesophageal reflux or recent upper airway tract infection; (3) had a risk of aspiration; or (4) had a body mass index greater than 35 kg/m^2^. Equal numbers of eligible patients were randomly allocated to the SLT group or DLT group by an investigator not involved in this study, using random numbers generated in Microsoft Excel 2010 (Microsoft Corp., USA). Patients were randomized to their study group in the operating room after airway assessment.

On arrival in the operating room, all patients were monitored using an electrocardiogram and pulse oximeter, and non-invasive blood pressure and bispectral index (BIS) were measured. After preoxygenation with 100% oxygen for 3 min, fentanyl 1.0–1.5 μg/kg and thiopental sodium 4.0–5.0 mg/kg were administered. On loss of consciousness, muscles were relaxed using rocuronium 0.6 mg/kg and anaesthesia was maintained with sevoflurane in oxygen. The thyromental distance mouth opening was measured 2 min after muscle relaxation, and the laryngeal view was evaluated with direct laryngoscopy according to the modified Cormack–Lehane classification. Mouth opening and modified Cormack–Lehane grade were rechecked after a semi-rigid foam neck collar (Philadelphia cervical collar) was placed on all patients. All tracheal intubations were performed with a flexible fiberoptic bronchoscope (PortaView^®^ LF-GP; Olympus Optical Company, Tokyo, Japan) with an outer diameter of 4.1 mm by the same investigator (DH Kim, an anaesthesiologist with over 20 years of experience with fiberoptic bronchoscope-guided intubation). In both groups, the polyvinyl chloride SLT or silicone DLT was preloaded before intubation. One investigator introduced the fiberoptic bronchoscope into the trachea via the patient’s mouth at the head of the bed, while the other performed jaw-thrust manoeuvre to clear the airway and provide sufficient space for fiberoptic bronchoscope passage. After confirming that the tip of the fiberoptic bronchoscope was on the carina, the preloaded tube was railroaded along the fiberoptic bronchoscope. If the railroading met resistance, the tracheal tube was pulled back 2–3 cm, rotated 90° counter-clockwise, and then reinserted. If the resistance was not resolved after this, the degree of counter-clockwise rotation was increased to more than 120° or clockwise rotation was attempted until successful intubation.

**Figure 1:**
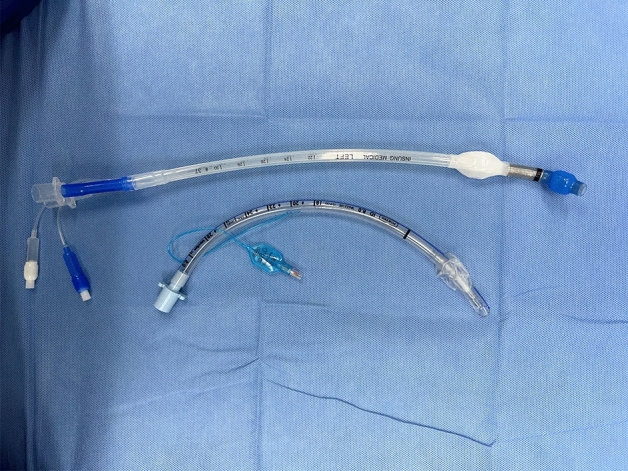
Real photo of silicone double-lumen tube and polyvinyl chloride single-lumen tube.

In the SLT group, a standard bevelled polyvinyl chloride Portex^®^ endotracheal tube (Smith Medical, Hythe, UK, Fig. 1) was used. The size of the endotracheal tube was chosen according to the sex of the patient: 7.0 mm internal diameter and 9.6 mm outer diameter SLT for females and 8.0 mm internal diameter and 10.9 mm outer diameter SLT for males in the SLT group. The preloaded polyvinyl chloride SLT was introduced along the fiberoptic bronchoscope (railroading over the fiberoptic bronchoscope) with the bevel of the tube oriented towards the patient’s left side. After intubation, the bronchial blocker (Coopdech Endobronchial Blocker Tube, Daiken medical Co., LTD, Japan) was inserted into the proper mainstream bronchus under fiberoptic bronchoscope control with a smaller fiberoptic bronchoscope (PortaView^®^ LF-GP; Olympus Optical Company, Tokyo, Japan; outer diameter 3.1 mm).

In the DLT group, a HumanBroncho^®^ silicone left-sided DLT (Insung Medical, Seoul, Korea, Fig. 1) was used according to the sex of the patient: size 35 Fr DLT (internal diameter short/long, 4.5 mm/7.0 mm; outer diameter short/long, 10.0 mm/13.3 mm) for female patients and 37 Fr DLT (internal diameter short/long, 4.9 mm/7.5 mm; outer diameter short/long, 10.5 mm/14.3 mm) for male patients. As described above, the fiberoptic bronchoscope was introduced into the trachea and then the preloaded silicone DLT was railroaded over the fiberoptic bronchoscope with the concave curvature facing left. The DLT was inserted into the left main bronchus until resistance was felt; then, the correct position of the DLT was confirmed by fiberoptic bronchoscope reinsertion through tracheal lumen after withdrawing it from the bronchial lumen.

All procedures were recorded by another investigator using a video camera for measurement of the following time points: (1) insertion time of fiberoptic bronchoscope, defined as the time from which the fiberoptic bronchoscope started to pass the tooth and reached above the carina; (2) railroading time, the time from positioning the fiberoptic bronchoscope above the carina until the tube was placed over the carina, with the tube not needing to be in the final position; (3) time to tracheal intubation, insertion time of fiberoptic bronchoscope plus the railroading time; and (4) total time for correct tube and bronchial blocker positioning, the time from which the fiberoptic bronchoscope started to pass the tooth till confirming the proper position of the DLT or bronchial blocker for adequate OLV. The difficulty of railroading was evaluated by the investigator (DH Kim) as follows: (grade I) the railroading was easily done following the natural curvature of the SLT or DLT; (grade II) failure of grade I railroading, necessitating tube readvancement after 90° counter-clockwise rotation; (grade III) failure of grade II railroading, requiring another manipulation like 120° counter-clockwise rotation, 90° clockwise rotation, rerotation, or external manipulation; (grade IV) failure of grade III railroading, needing direct laryngoscopy or removal of anterior cervical neck collar. If the time to tracheal intubation was over 120 s, it was recorded as a failed case and intubation was performed with another method after removing neck collar.

In the post-anaesthesia care unit (PACU) after surgery, the incidence of sore throat, difficulty in swallowing, and hoarseness were evaluated by an investigator who was not involved in the study.

The primary outcome of this study was the time to tracheal intubation between the two groups, designed as a non-inferiority test. The sample size required for non-inferiority was calculated based on an assumed standard deviation of 18 s with a non-inferiority margin of 10 s^9^. A sample size of 40 patients per group was obtained with an alpha error of 0.05 and 80% power. The secondary outcomes include the time to insert the fiberoptic bronchoscope, the railroading time, the total time for correct tube and bronchial blocker positioning, the difficulty of railroading over a fiberscope bronchoscope, and the incidence of sore throat, hoarseness, and swallowing difficulties.

Statistical analysis was performed using SPSS version 21 (SPSS Inc., Chicago, IL, USA). Continuous data between groups were analysed using Student’s t-test or Mann–Whitney U-test as appropriate. Normality of the data distribution was tested using the Kolmogorov–Smirnov test, and results are presented as means with standard deviations and medians with interquartile ranges (IQR) as appropriate. Categorial data between groups were analysed using Chi-square or Fisher’s exact tests as appropriate. Results are presented as numbers and percentages. Continuous and categorial data before and after applying neck collar were analysed using paired t-test or Wilcoxon signed-rank test. A *p* value of < 0.05 was considered statistically significant. To demonstrate non-inferiority of the primary outcome, the primary outcome was assessed by a two-sided 95% confidence interval using Hodges–Lehmann method. If the upper bound of the 95% confidence interval in the time to endotracheal tube intubation between groups was below the non-inferiority margin of 10 s, non-inferiority would be declared.

## Results

**Figure 2:**
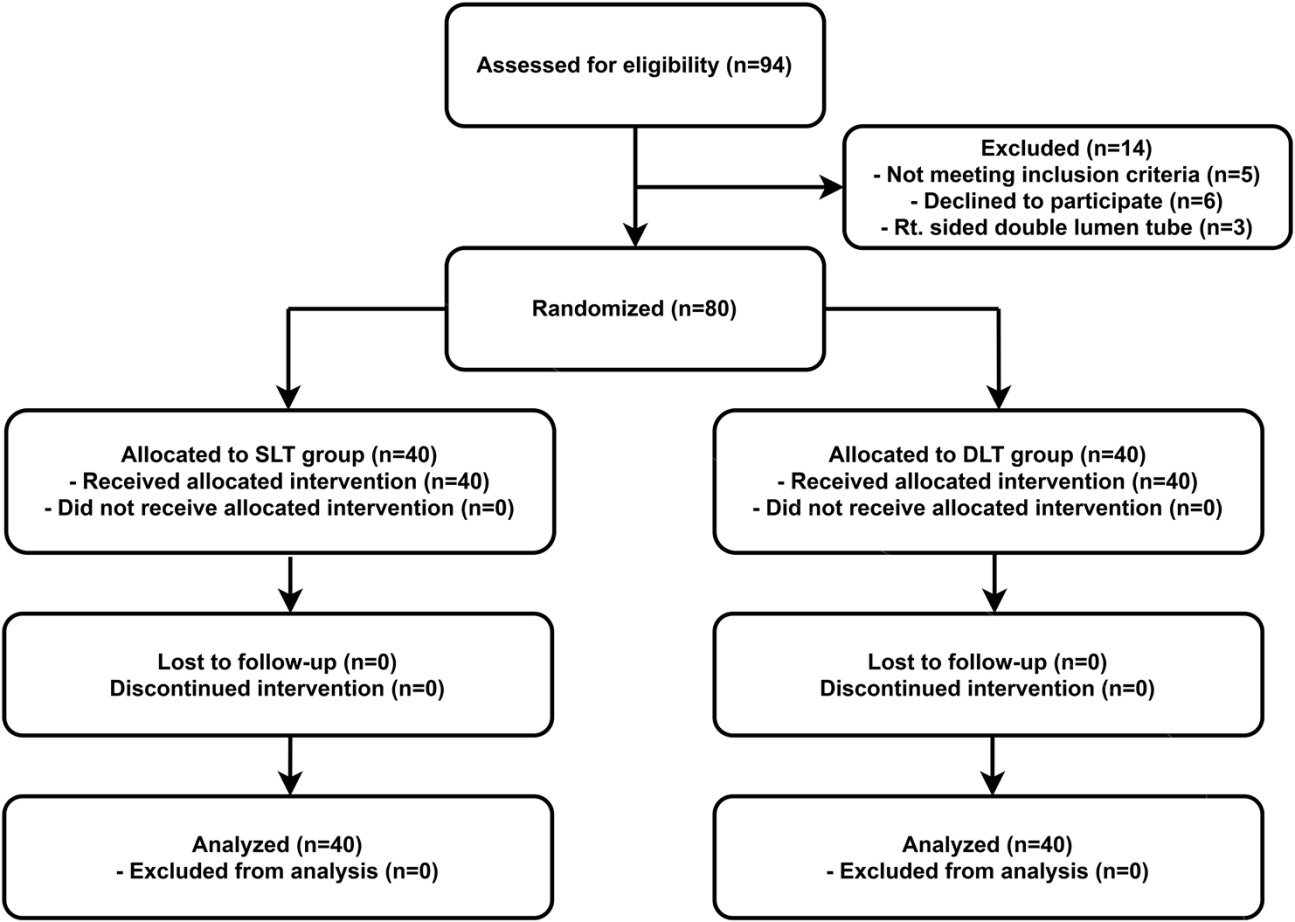
CONSORT flow diagram of recruitment and assessment of study participants.

Out of 94 patients assessed for eligibility, 80 were enrolled in this study and randomized into two groups. The CONSORT flowchart for this study is presented in Fig. 2. Tracheal intubation using the fiberoptic bronchoscope was successful in all patients on the first attempt. Patient characteristics and airway assessment data showed no significant difference between groups (Table 1). The data reflecting the effect of the neck collar application on airway showed significant changes. The mouth opening was significantly reduced after semi-rigid neck collar application (*p* < 0.001) and modified Cormack–Lehane grade as assessed using a direct laryngoscope had significantly worsened (*p* < 0.001), but there was no difference between groups. The hemodynamic variables were appropriately managed by the discretion of another anesthesiologist.

**Table 1:** Patient characteristics and airway assessment results.

Variables	SLT group (n = 40)	DLT group (n = 40)
Sex (M/F)	16/24	18/22
Age (years)	45 ± 16	50 ± 15
Weight (kg)	67 ± 14	67 ± 10
Height (cm)	168 ± 9	166 ± 8
ASA PS (I/II)	28 / 12	25 / 15
Thyromental distance (cm)	5.9 ± 1.0	6.0 ± 0.7
Mouth opening-before neck collar (cm)	4.6 ± 0.6	4.3 ± 0.6
Mouth opening-after neck collar (cm)	2.1 ± 0.3	2.1 ± 0.4
C-L grade by direct laryngoscope		
I	25	17
IIa	7	5
IIb	6	13
III	2	5
IV	0	0
C-L grade by direct laryngoscope after neck collar		
I	0	0
IIa	0	0
IIb	13	9
III	23	23
IV	4	8

Data are represented as mean (standard deviation) or number. *SLT Group* tracheal intubation over fibreoptic bronchoscope with polyvinyl single-lumen tube, *DLT Group* tracheal intubation over fibreoptic bronchoscope with silicone double-lumen tube, ASA PS American society of Anaesthesiologist physical status, *C-L grade* modified Cormack-Lehane grade.

The airway management data are presented in Table 2. There was no patient with a failed intubation time to tracheal intubation (over 120 s). The median time to tracheal intubation was 22 s in both the SLT and DLT groups, and the median difference and 95% confidence intervals were 1 s and − 2 to 4 s, respectively. Since the upper confidence boundary was positioned below the pre-specified non-inferiority margin of 10 s, non-inferiority was declared. The insertion time for the fiberoptic bronchoscope was similar between the two groups (median time, 12 vs 12 s; median difference, − 1 s; 95% confidence interval, − 2 to 1 s; *p* = 0.420). The railroading time was significantly longer in the SLT group than in the DLT group (median time, 11 vs 10 s; median difference, 2 s; 95% confidence interval, 0 to 3 s; *p* = 0.030). The difficulty of railroading over the fiberoptic bronchoscope (graded on four-point scale) was significantly higher in the SLT group compared with that in the DLT group (p < 0.001). The total time taken for correct tube and bronchial blocker positioning was longer in the SLT group than in DLT group (median time, 78 vs 35 s; median difference, 46 s; 95% confidence interval 41 to 31 s; *p* < 0.001). There were no significant differences in sore throat, difficulty in swallowing, and hoarseness between two groups, as assessed in the PACU.

**Table 2:** Airway management data.

	SLT Group (n = 40)	DLT Group (n = 40)	Median difference DLT-SLT (95% CI)	*P*-value
FOB insertion time (s)	12 [9–15]	12 [7–12]	− 1 (− 2 to 1)	0.420
Railroading time (s)	11 [10–13]	10 [7–12]	2 (0 to 3)	0.030
Time to tracheal intubation (FOB insertion time plus railroading time; s)	22 [19–30]	22 [18–27]	1 (− 2 to 4)	0.467
	The upper confidence boundary was below the prospectively determined non-inferiority margin of 10 s: non-inferiority			
Total time for correct tube and bronchial blocker positioning (s)	78 [74–96]	35 [28–44]	46 (41 to 31)	< 0.001
Difficulty of railroading (I/II/III/IV)	10/27/3/0	27/12/1/0		< 0.001
In PACU				
Sore throat (Y/N)	4/36	4/36		0.644
Difficulty swallowing (Y/N)	3/37	5/35		0.712
Hoarseness (Y/N)	4/36	6/34		0.737

Data are represented as median [interquartile range] or number. *SLT Group* tracheal intubation over fibreoptic bronchoscope with polyvinyl single-lumen tube, *DLT Group* tracheal intubation over fibreoptic bronchoscope with silicone double-lumen tube, *FOB* fiberoptic bronchoscope, *PACU* post anesthesia care unit.

## Discussion

This study demonstrated that the time to tracheal intubation with the silicone DLT over a fiberoptic bronchoscope is non-inferior to the use of a polyvinyl chloride SLT over a fiberoptic bronchoscope in patients with semi-rigid neck collar. Moreover, the silicone DLT showed significantly better performance in terms of railroading time and the difficulty of railroading than the polyvinyl chloride SLT.

Some studies showed that direct intubation with the DLT over a fiberoptic bronchoscope would be more difficult than the same for SLT^9–13^. Only two case reports showed that direct intubation with a polyvinyl chloride DLT over the fiberoptic bronchoscope was actually implemented in the awake state or using a shortened DLT^5,6^. Our previous study results demonstrated that direct tracheal intubation with the silicone DLT using the fiberoptic bronchoscope was fast and had a similar performance as that of polyvinyl chloride SLT^8^. Since this result was obtained after assessment on patients with a normal airway, there was uncertainty about whether the silicone DLT over a fiberoptic bronchoscope could also be useful in patients with difficult airways. In this study, we simulated a difficult airway by applying a semi-rigid neck collar to the patients. After application of the neck collar, mouth opening was reduced by more than half and modified Cormack–Lehane grade became worse. The time to tracheal intubation, which included the insertion time of fiberoptic bronchoscope, and the railroading time, indicating time for securing airway, was similar between the SLT and DLT groups. The railroading time and the difficulty of railroading were significantly better in the DLT group than in those in the SLT group. In other words, the silicone DLT was easier to use than the polyvinyl chloride SLT for securing the airway using a fiberoptic bronchoscope. However, the simulated difficult airway by cervical neck collar could not reflect the full range of clinically challenging airways. In particular, if the airway is narrowed due to swelling or pressing, it may be difficult for DLT to pass, so there may be a limit to DLT selection. However, unless the size of the DLT itself is a problem, the silicone DLT could be a feasible option as a first choice in tracheal intubation using fiberoptic bronchoscope for patients with an anticipated difficult airway who need lung ventilation.

As such, the conventional stereotype that a large diameter, long length, and rigid characteristics of the DLT would act as a disadvantage during intubation did not have a significant impact on this study using a silicone DLT^2,4,14^. We presumed that this was due to the flexibility of silicone material and the shape of the tip. The silicone DLT is light and flexible; therefore, it was well mounted on the fiberoptic bronchoscope and could be easily manipulated. During railroading, the body of the silicone DLT adapted flexibly to the curvature of the trachea. Therefore, even if the length of tube was long, once the tip passed the vocal cord, the remaining part of the tube also went smoothly without resistance. The tip of the silicone DLT was also flexible and had an oval shape with an obtuse angle, which might be a contributing effect for reducing impingement^15^. The biggest concern was the large diameter of the silicone DLT as smaller size tubes have been preferred for their ease of insertion in patients with difficult airway intubation^14,15^.

To date, the part that is considered to have a large diameter in the silicone DLT is the body of the tube, not the tip diameter. Unlike the polyvinyl chloride SLT, which has the same body and tip diameter, the tip diameter of the silicone DLT is similar to or even smaller than the tip diameter of the polyvinyl chloride SLT. For female patients, the outer diameter of the tip was only 0.4 mm larger in silicone DLT than in polyvinyl chloride SLT, and for male patients, rather it was smaller by 0.4 mm in silicone DLT. In addition, the non-bevelled, oval shape of the tip with an obtuse angle might be also a contributing factor for smooth railroading^15^. Taken together, these factors are the reasons that the DLT was not difficult to insert and did not have any effect in case of intubation with silicone DLT over a fiberoptic bronchoscope in this study.

In addition, the whole process was simpler and less time consuming when using silicone DLT as the process of inserting and positioning of bronchial blockers for OLV was omitted in the silicone DLT group and replaced by the process of confirming proper position through a tracheal lumen of the silicone DLT. Therefore, the total time for correct tube placement was significantly shorter in the silicone DLT group than in the SLT group.

This study had several limitations. First, the fiberoptic bronchoscope insertion was performed by one investigator. Thus, the results cannot be generalized to less-experienced investigators, especially regarding time taken. Second, the factors that have not been mentioned in the discussion, such as tube material or stiffness, may have been involved in the railroading of the DLT. For additional clarity, it will be necessary to conduct more detailed research in the future. Third, potential bias could exist because the investigator was not blinded to the group allocation. Fourth, the simulated difficult airway using a cervical neck collar could not actually reflect a clinically difficult airway. Further, patients with excessive secretions of blood or sputum or oedematous airway were not included in our study. This might particularly be a problem in the situation of a limiting jaw thrust manoeuvre like facial trauma because, in our study, it was an essential manoeuvre for securing oral space.

In conclusion, tracheal intubation over a fiberoptic bronchoscope with a silicone DLT can be as quick as that using a polyvinyl chloride SLT; it can also be performed with less difficulty than that using SLT, suggesting that this could be a feasible option in patients with expected difficult airway requiring lung separation.

## Data Availability

The data are not available for public access because of patient privacy concerns but are available from the corresponding author on reasonable request approved by the Ajou institutional review boards.
